# *Fusobacterium nucleatum*: a novel immune modulator in breast cancer?

**DOI:** 10.1017/erm.2023.9

**Published:** 2023-04-03

**Authors:** Alexa Little, Mark Tangney, Michael M. Tunney, Niamh E. Buckley

**Affiliations:** 1 School of Pharmacy, Queen's University Belfast, Belfast, Northern Ireland, UK; 2 Cancer Research, University College Cork, Cork, Ireland; 3 APC Microbiome Ireland, University College Cork, Cork, Ireland

**Keywords:** Breast cancer, drug resistance, *F. nucleatum*, inflammation, tumour microenvironment

## Abstract

Breast cancer was the most commonly diagnosed cancer worldwide in 2020. Greater understanding of the factors which promote tumour progression, metastatic development and therapeutic resistance is needed. In recent years, a distinct microbiome has been detected in the breast, a site previously thought to be sterile. Here, we review the clinical and molecular relevance of the oral anaerobic bacterium *Fusobacterium nucleatum* in breast cancer. *F. nucleatum* is enriched in breast tumour tissue compared with matched healthy tissue and has been shown to promote mammary tumour growth and metastatic progression in mouse models. Current literature suggests that *F. nucleatum* modulates immune escape and inflammation within the tissue microenvironment, two well-defined hallmarks of cancer. Furthermore, the microbiome, and *F. nucleatum* specifically, has been shown to affect patient response to therapy including immune checkpoint inhibitors. These findings highlight areas of future research needed to better understand the influence of *F. nucleatum* in the development and treatment of breast cancer.

## Breast cancer

Breast cancer (BC) has exceeded lung cancer to become the most commonly diagnosed cancer worldwide, with 2.3 million cases in 2020 alone (Ref. 1). At present, 70–80% of early-stage, non-metastatic cases are curable (Ref. 2). However, secondary/metastatic BC is considered incurable with the currently available treatments. Unfortunately, in 2020 there were over 650 000 BC-related deaths worldwide, contributing to approximately 7% of cancer deaths that year (Ref. 1). Therefore, there is an unmet clinical need to understand what causes certain cancers to resist treatment and what drives metastasis.

BC is a heterogeneous disease showing molecular and histological diversity between patients, resulting in variability in disease outcome and response to treatment. Biomarker expression has been used successfully to stratify breast tumours into molecular subgroups, guide treatment options and to develop targeted treatments such as endocrine therapies. The current molecular biomarkers with clinical significance include the oestrogen receptor (ER*α*), progesterone receptor (PR) and human epidermal growth factor receptor 2 (HER2) (Ref. 2). Additionally, BCs that are ER*α*/PR negative and lack HER2 amplification are grouped as triple negative breast cancers (TNBCs), which lack available targeted treatment options (Ref. 3), although some advances are being made in subsets of TNBC through the use of immune checkpoint inhibitors (ICIs) (Refs 4, 5, 6, 7) and/or antibody-drug conjugates (Ref. 8).

However, there are still limitations with current BC treatments, where patients may relapse even with subtype-specific treatment regimens. Therefore, further stratification and the identification of more effective and actionable prognostic and predictive biomarkers are required to improve patient management.

This review aims to examine the known molecular consequences of the species of bacteria *Fusobacterium nucleatum* (*F. nucleatum*) within the tumour microenvironment (TME), potentially identifying actionable pathways modulated by the bacterium that may have relevance in the BC setting.

## The microbiome and cancer

The human body is host to a large population of microbes, estimated at 10–100 trillion cells (Ref. 9), the majority of which exist within the gastrointestinal (GI) tract. Due to the development of next-generation sequencing techniques, organs which were previously believed to be sterile have been revealed to host microbial populations (Ref. 10). Furthermore, the human microbiome is shaped via co-evolution with the host, resulting in large compositional variations between age, sex, diet and geographical location. Therefore, the microbiome may contribute to the diversity observed in disease outcomes and treatment response between patients.

The imbalance in the relationship between the host and the microbiota (dysbiosis) is characterised by a reduction in the diversity of microbes present, and a shift towards a population in which pathogenic bacteria dominate. With the microbiome recently included as a hallmark of cancer (Ref. 11) growing evidence suggests that both cancer-protective and tumour-promoting species exist, and can influence susceptibility, development, therapeutic response and metastasis (Ref. 12) of certain cancers. Therefore, particular members of the microbiome could be, and have already been, identified as biomarkers with clinical importance, including the human papilloma virus (HPV), hepatitis B and C and the bacterium *Helicobacter pylori* (Ref. 13).

However, more microbial species have been identified in recent years within tumour tissue as a result of the development of high-depth next-generation sequencing of bacterial 16S ribosomal RNA and more complete databases of sequenced organisms (Refs 13, 14, 15, 16, 17, 18). Critically, these approaches have been expanded to also characterise low-biomass intra-tumoural microbiomes, including introducing stringent pipelines which account for background noise and contamination (Ref. 10), and mining shotgun sequencing data generated on tumour tissue biopsies (Ref. 19).

A number of these newly detected intra-tumoural microbes have been shown to modulate or contribute to cancer (Ref. 20). Conversely, some species have been exploited for cancer treatments such as probiotic treatments given alongside conventional therapy regimes or bacteria-assisted tumour-targeting therapies (Refs 21, 22).

Importantly, in a study by Nejman *et al*. (Ref. 10) which characterised the link between the microbiome and different types of solid tumours using next-generation sequencing, breast tumours were shown to have a rich and more diverse microbiome compared to the other tumour types tested, including melanoma and lung, but not including the GI tract. Furthermore, they noted variation within the dominant bacterial taxa between the ER*α*+, PR+ and HER2+ subtypes of BC (Ref. 11). Other studies have confirmed that there is an altered microbiome in breast tumours compared with healthy tissue (Refs 23, 24, 25, 26, 27, 28, 29, 30), the findings of which have been reviewed previously (Refs 31, 32). The potential to utilise the bacterial signature of breast biopsy tissue to infer malignancy status has also recently been reported (Ref. 33).

Breast cancer-associated bacteria have been found predominantly to reside intracellularly, both within breast tumour epithelial cells and immune cells (Refs 10, 34). However, the microbiome of distant organs such as those of the GI tract can also affect carcinogenesis and progression of BC by influencing factors such as diet, obesity, levels of free circulating oestrogens and immune modulation (Refs 12, 35, 36). Moreover, the microbiome of both distant organs and the site of the tumour has been linked to local and systemic impacts on cancer chemotherapy efficacy and toxicity (Refs 12, 37). Studies have also shown that modulating the gut microbiome before and during chemotherapy treatment could improve efficacy and reduce the incidence of adverse events (Refs 38, 39), and more specifically, the gut microbiome was used as a predictive biomarker for doxorubicin responsiveness in a 4T1 murine TNBC model (Ref. 37).

**Figure 1.**
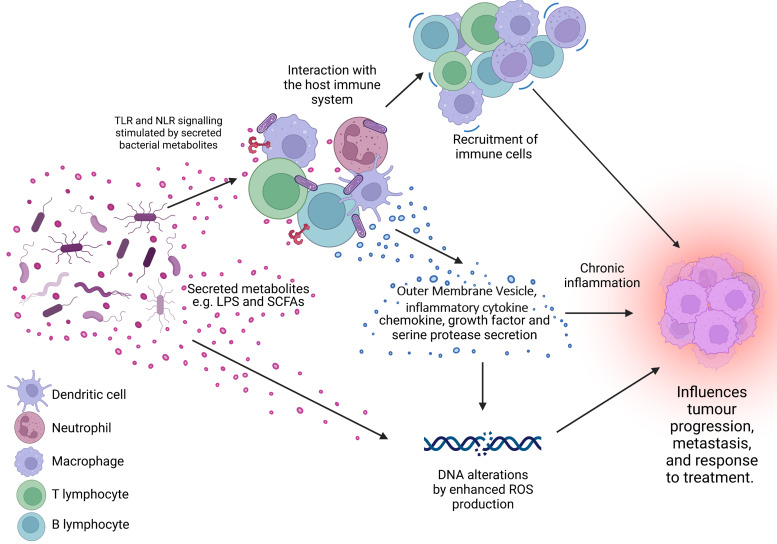
The microbiome is a key regulator of the tumour microenvironment (TME). Secreted factors and ‘immunomodulatory’ factors produced by bacteria can activate damage sensors on immune cells, for example, outer membrane vesicles which contain proinflammatory molecules such as lipopolysaccharide (LPS) on Gram-negative bacteria which stimulates Toll-like receptor (TLR)-4 signalling in immune cells. This activation results in the expression of a range of chemokines and cytokines, which further influence the recruitment and behaviour of immune cells within the TME and can lead to a state of chronic inflammation. Cells present in the TME can also produce growth factors and serine proteases which induce tumour progression. Furthermore, bacteria secrete metabolites such as short chain fatty acids (SCFAs) which can interact with the TME to reshape it, and/or cause genomic instability within the cells. LPS, lipopolysaccharide; SCFA, short-chain fatty acid; ROS, reactive oxygen species; TLR, Toll-like receptor; NLR, Nod-like receptor. Figure created with BioRender.

Furthermore, some bacterial species have been shown to alter the TME, which is important in tumour formation, progression, metastasis and drug resistance (Refs 40, 41). Bacterial colonisation of the tumour has been shown to activate the intertwined processes of tumour-promoting inflammation and evasion of tumour destruction by the immune system (Fig. 1) (Refs 11, 42). Investigations into how the intra-tumoral bacteria may influence the breast TME are only beginning. However, remodelling of the TME in BC by bacteria has already been shown using the 4T1 syngeneic model inoculated with *Escherichia coli K-12*, where increased type IV collagen deposition, increased matrix metalloproteinase 9 (MMP9) expression and altered distribution of tumour-associated macrophages were observed (Ref. 24). Additionally, intraductal injection of mouse teats with *Bacteroides fragilis* resulted in increased local inflammation, tissue fibrosis and higher T-cell infiltration than in control mice (Ref. 43).

## *Fusobacterium nucleatum*: an overview

*F. nucleatum* is a Gram-negative, anaerobic, adhesive bacterium and is commonly found within the oral mucosa where it aids in biofilm formation, supporting a normal oral microenvironment (Ref. 44). However, *F. nucleatum* has also been associated with adverse pregnancy outcomes (Refs 45, 46), appendicitis (Ref. 47) and importantly, many tumour types (Refs 10, 48, 49). For example, *F. nucleatum* has been reported to be a potential biomarker for populations of colorectal cancer (CRC) (Refs 50, 51, 52, 53).

Studies have shown that *F. nucleatum* presence in tumour tissue is associated with poor overall survival (OS) in oesophageal squamous cell carcinomas (ESCC), early-stage HPV-negative tongue cancer (Ref. 54), as well as increased metastasis in CRC patients (Refs 52, 55, 56, 57, 58). However, in oral squamous cell carcinoma (OSCC), *F. nucleatum* presence is associated with a lower recurrence rate, reduced metastases and longer OS (Ref. 59). This highlights the complexity of host–pathogen relationships, and therefore the need for individual, context-specific studies.

Methods to detect and quantify specific microbes have advanced, and the development of RNA *in situ* hybridisation (Refs 60, 61, 62), next-generation sequencing (Refs 10, 49) and qPCR on tumour tissue (Refs 48, 63) has enabled detection of *F. nucleatum* in both high- and low-biomass tumour tissues.

*F. nucleatum* was identified in approximately 30% of breast tumours by Nejman *et al*. (Ref. 10), and within other BC cohorts (Refs 23, 29, 64, 65, 66). Additionally, while the abundance of *F. nucleatum* relative to cancer cells is low, it is shown to increase in abundance in higher stage breast tumours (Ref. 28). However, the clinical significance has not yet been fully elucidated for *F. nucleatum* in the breast. Given the findings that *F. nucleatum* is associated with both favourable outcomes in OSCC, and adverse outcomes in CRC and ESCC, it will be important in the future to determine the significance of *F. nucleatum* in the breast on survival outcomes.

Parhi *et al*. (Ref. 64) showed that *F. nucleatum* promoted mammary tumour growth and, critically, metastatic progression when inoculated into mice. They suggested that this effect may be mediated by suppression of T-cell infiltration into the TME and/or increased expression of MMP9 (Ref. 64).

## The oncogenic mechanisms of *F. nucleatum* in cancer

**Figure 2.**
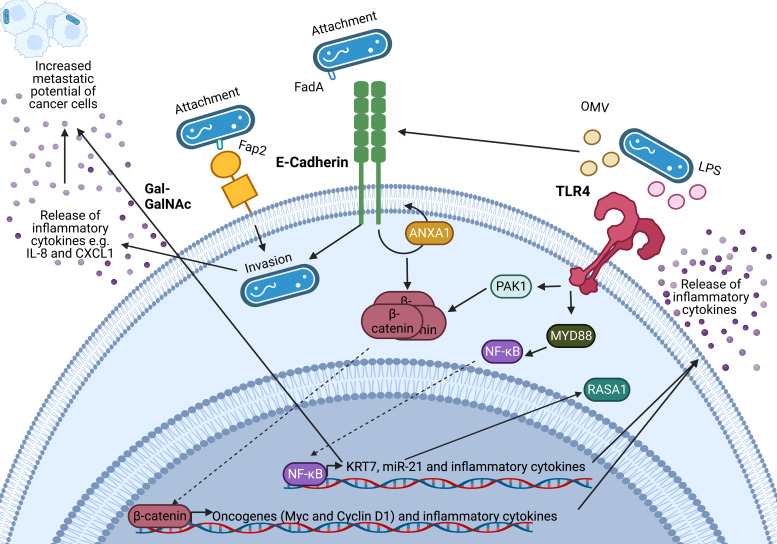
Known oncogenic pathways modulated by *Fusobacterium nucleatum*. *F. nucleatum* (shown in blue) binds to tumour cells via interaction of its Fap2 protein with D-galactose-*β*(1–3)-N-acetyl-D-galactosamine (Gal-GalNAc) or by FadA interacting with E-cadherin, which is enhanced by Annexin A1 (ANXA1), enabling attachment and invasion of tumour cells. *F. nucleatum* also secretes outer membrane vesicles (OMVs) and lipopolysaccharide (LPS) which interact with the Toll-like receptors (TLRs) to initiate downstream signalling pathways that mediate the release of inflammatory cytokines and transcription of miR-21 which is known to regulate the activity of the oncoprotein RASA1. The E-cadherin and TLR4 signalling induced by *F. nucleatum* binding stimulates *β*-catenin accumulation in the cytoplasm and its subsequent translocation to the nucleus where it upregulates transcription of oncogenes including c-MYC and Cyclin D1. Furthermore, *F. nucleatum* is able to aid metastasis through OMV-mediated degradation of E. cadherin, NF-*κ*B mediated increased expression of keratin 7 (KRT7), and via induction of the inflammatory cytokines IL-8 and CXCL1. Figure created with BioRender.

An important feature of *F. nucleatum* is its ability to bind to a variety of host and neighbouring bacterial cells via a range of virulence factors including the Fap2 protein that binds to the sugar D-galactose-*β-*N-acetyl-D-galactosamine (Gal-GalNAc) (Refs 1, 2, 3)(Refs 64, 67) which is overexpressed in CRC and BC (Refs 64, 67). Specifically, *F. nucleatum* binds to tumour cells, influencing downstream oncogenic and pro-metastatic signalling (Refs 68, 69, 70, 71, 72, 73, 74). A summary of known oncogenic *F. nucleatum* interactions in CRC through *F. nucleatum* virulence factors is summarised in Figure 2 (Refs 73, 75, 76, 77, 78, 79). This review expands on the influence of *F. nucleatum* on the TME, and how these findings may guide the research into the relationship between BC and *F. nucleatum.*

### *Fusobacterium nucleatum* and inflammation within the tumour microenvironment

Inflammation is one of the hallmarks of cancer, with up to 20% of cancers being preceded by chronic inflammation at the site (Refs 80, 81). While *F. nucleatum* can bind to cancer cells and activate oncogenic signalling directly, as observed in CRC, there is also evidence that *F. nucleatum* is able to indirectly promote tumour progression by modulating the inflammatory microenvironment.

*F. nucleatum* infection is closely linked to NF-*κ*B signalling by numerous studies in multiple cell types (Refs 63, 73, 74, 82, 83, 84, 85, 86), however this link has not yet been investigated in BC. NF-*κ*B signalling can be activated by bacteria through immune receptors including the Toll-like receptors (TLRs) to upregulate many chemokines and cytokines (described in further detail below). For example, TLR2 and TLR4 are implicated in *F. nucleatum*-stimulated macrophage cytokine production (Ref. 87). Constitutive activation of NF-*κ*B signalling has been linked to inflammation and cancer (Ref. 88) *via* regulation of genes involved in cell proliferation, differentiation and innate and adaptive immune responses (Ref. 89).

A number of studies have identified an inflammatory signature associated with *F. nucleatum* presence within CRC (Refs 67, 79, 85, 90). Specifically, *F. nucleatum* presence within human colonic tumours has been associated with the upregulation of the pro-inflammatory cytokines IL-6, IL-8 and IL-1*β*, among others (Refs 79, 85, 90). It is possible that with further investigation into the breast TME, comparisons could be made between the effect of *F. nucleatum* in these two cancers.

In BC, upregulation of serum IL-6 levels is associated with poor prognosis (Refs 91, 92), where hormone-sensitive tumour cells have a greater response to IL-6 (Ref. 93). IL-6 has been linked to epithelial-mesenchymal transition (EMT) in BC and enhances mesenchymal stem cell recruitment in the breast TME (Refs 94, 95). Therefore, it is interesting that IL-6 secretion is induced by *F. nucleatum* infection in B lymphocytes (Ref. 96) and macrophages (Ref. 83). Similarly, in CRC, Wang *et al*. noted that *F. nucleatum* infected CRC cells displayed an EMT cancer stem cell-like behaviour as a result of IL-6/STAT3 signalling (Ref. 97).

Additionally, multiple studies have identified upregulated IL-8 as a result of *F. nucleatum* infection in CRC cells (Refs 68, 79, 85, 96, 98). IL-8 in BC is associated with positive lymph node status and higher-stage tumours (Refs 99, 100).

**Figure 3.**
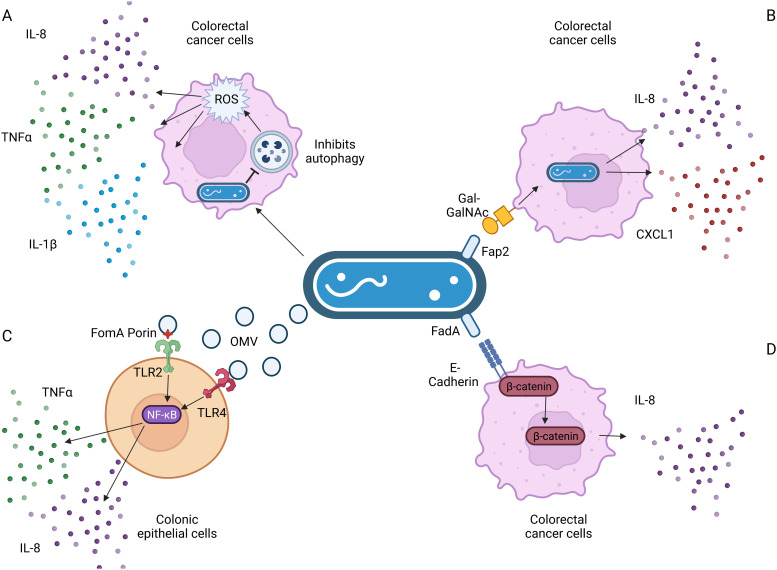
Known pathways induced by *F. nucleatum* binding that result in increased interleukin-8 (IL-8) secretion. (a) *F. nucleatum* infection in Caco-2 colorectal cancer cells impaired autophagic flux, which enhanced the production of TNF-*α*, IL-1*β* and IL-8 via the increase in reactive oxygen species (ROS). (b) *F. nucleatum* binding via its FadA adhesin to the sugar D-galactose-*β*(1–3)-N-acetyl-D-galactosamine (Gal-GalNAc) on colorectal cancer cells enables invasion, which further stimulates the release of IL-8 and CXCL1. (c) Outer membrane vesicles and the porin FomA secreted by *F. nucleatum* stimulate Toll-like receptors (TLRs) 2 and 4 on colonic epithelial cells, inducing NF-*κ*B signalling that results in increased IL-8 secretion. (d) *F. nucleatum*'s FadA adhesin binds to E-cadherin, activating *β*-catenin signalling in CRC cells, resulting in increased expression of pro-inflammatory cytokines, including IL-8. Figure created with BioRender.

In colonic cells, *F. nucleatum-*secreted outer membrane vesicles, and the FomA porin that is present on them, induced IL-8 expression in a TLR2- and TLR4-dependent manner (Refs 96, 101), as a result of NF-*κ*B signalling (Ref. 102). TLRs recognise microbial products, such as lipopolysaccharide from Gram-negative bacteria like *F. nucleatum* and stimulate secretion of inflammatory mediators and/or activate immune cells. Extracellular vesicles were further found to induce IL-8 secretion in colonic epithelial cells in a TLR4-dependent mechanism (Ref. 101), again involving NF-*κ*B signalling. *F. nucleatum* induces IL-8 expression through pathways involving increased reactive oxygen species (Ref. 103), *β*-catenin signalling (Refs 73, 75) and invasion via its FadA adhesin (Ref. 67), as depicted in Figure 3.

### *Fusobacterium nucleatum* and the tumour immune microenvironment

The studies highlighted in Table 1 provide abundant evidence that *F. nucleatum* is capable of altering the composition and actions of the immune cell population of the TME. It is possible that *F. nucleatum* promotes an immunosuppressive TME, enabling tumour cell escape from immune surveillance. While research into how the presence of *F. nucleatum* alters the immune response to other cancers is more advanced, little is known at this time with respect to the impact of *F. nucleatum* on the TME in BC. Given the importance of the immune response to BC and its impact on survival, drug efficacy and metastatic potential (Ref. 104), the presence of *F. nucleatum* and its known ability to alter the tumour immune microenvironment is an important area of future research.

**Table 1.** The effect of *F. nucleatum* on immune cells from different studies

Cell type	Model	Effect of *F. nucleatum*	Mechanism	Ref
Peripheral blood lymphocytes	Human peripheral blood lymphocyte cells	Inhibition	Via altered DNA, RNA and protein synthesis	(Ref. 105)
	Human peripheral blood mononuclear cells	Reduction	Induction of apoptotic cell death	(Ref. 106)
CD3 + T lymphocytes	Human T lymphocyte cell line	Inhibition of replication	Prevented from entering the G0/G1 phase of cell cycle	(Ref. 107)
	Human T lymphocyte cell line	Reduction	Cell death induced via Fap2 and RadD proteins	(Ref. 108)
	Human CRC tumour tissue	Reduction	Unknown	(Ref. 109)
CD4 + T lymphocytes	Murine CRC model	No change	Unknown	(Ref. 85)
	Human CRC tumour tissue	Reduction	Via a reduced expression of T lymphocyte developmental protein TOX	(Ref. 110)
	CRC lymphocyte cell line	Inhibition	The interaction of the human TIGIT and Fap2	(Ref. 111)
	Human CD4+ cells	Inhibition	*F. nucleatum* activates CEACAM1	(Ref. 112)
	Human CD4+ cells	Inhibition	*F. nucleatum* binds to and activates CEACAM1 via CbpF	(Ref. 113)
	Murine BC model	Reduction	Unknown	(Ref. 64)
	Human OSCC tumour tissue	Reduction	Unknown	(Ref. 59)
T-regulatory lymphocytes (TREGS)	Human ESCC tumour tissue	Increase	Unknown	(Ref. 62)
	Human intestine tissue and mouse models	Increase	*F. nucleatum* stimulates Toll-like receptors 2 and 4	(Ref. 114)
TH17T lymphocytes	Murine CRC model	Increase	Via a FFAR2 (SCFA receptor) dependent manner	(Ref. 115)
CD8+ T lymphocytes	Murine CRC model	No change	Unknown	(Ref. 85)
	CRC lymphocyte cell line	Inhibition	The interaction of the human TIGIT and Fap2	(Ref. 111)
	Human CD8+ cells	Inhibition	*F. nucleatum* activates CEACAM1	(Ref. 112)
	Murine BC model	Reduction	Unknown	(Ref. 64)
	Human ESCC tumour tissue and cell line	Inhibition	*F. nucleatum* stimulates the CD8+ cell surface inhibitory receptor KIR2DL1 expression	(Ref. 116)
B lymphocytes	Human OSCC tumour tissue	Reduction	Unknown	(Ref. 59)
Natural killer cells	Murine model	Reduced colonic NK cell activity and frequency	Unknown	(Ref. 117)
	CRC natural killer cell line	Inhibition	The interaction of the human TIGIT and Fap2	(Ref. 111)
	Human NK cells	Inhibition	*F. nucleatum* activates CEACAM1	(Ref. 112)
Macrophages	Human OSCC tumour tissue	Reduction in M2 macrophages	Unknown	(Ref. 59)
	Mouse and human CRC tumour tissue and cultured macrophages	Promotes M2 polarisation	via a TLR4/IL-6/p-STAT3/c-MYC pathway	(Ref. 83)
	Human CRC tumour tissue	Increase	Unknown	(Ref. 118)
	Human CRC tumour tissue and patient faeces	Increased macrophage infiltration and M2 polarisation	Via CCL20 activation	(Ref. 119)
	Human CRC tumour tissue	Promotes M2 polarisation	*F. nucleatum* activates the TLR4/NF-*κ*B/S100A9 cascade	(Ref. 120)
	Macrophage cell line	Promotes M1 polarisation	AI-2 activates the TNFSF9/IL-1*β* pathway	(Ref. 121)

AI-2; autoinducer-2, BC; breast cancer, CbpF; chlorine-binding protein; CCL20, chemokine (C-C motif) ligand 20; CD, cluster of differentiation; CEACAM1, CEA cell adhesion molecule 1; c-MYC, cellular-MYC; CRC, colorectal cancer; DNA, deoxyribonucleic acid; ESCC, oesophageal squamous cell carcinoma; FFAR2, free fatty acid receptor 2; IL-1*β*, interleukin 1*β*; IL-6, interleukin-6; KIR2DL1, killer cell immunoglobulin-like receptor 2DL1; NF-*κ*B, nuclear factor kappa B; NK, natural killer cell; OSCC, oral squamous cell carcinoma; p-STAT3, phospho-signal transducer and activator of transcription 3; RNA, ribonucleic acid; SCFA, short-chain fatty acid; S100A9, S100 calcium-binding protein A9; TIGIT, T-cell immunoreceptor with Ig and ITIM domains; TLR4, Toll-like receptor 4; TNFSF9, tumour necrosis factor ligand superfamily member 9; TOX, thymocyte selection-associated high mobility group box protein.

### *Fusobacterium nucleatum* and tumour response to treatment

Treatment of BC is multi-faceted, using a combination of surgery, radiotherapy and/or systemic therapy guided by the cancer molecular subtype (Ref. 2). However, drug resistance (intrinsic and acquired) often develops. *F. nucleatum* may influence treatment response in CRC, ESCC, OSCC and rectal adenocarcinoma. Given the presence of *F. nucleatum* in approximately 20% of BCs (Ref. 10), the importance of *F. nucleatum* as a biomarker which may aid in predicting response of BC subtypes to their treatments warrants further investigation. Additionally, *F. nucleatum* itself presents a potential therapeutic target, with antibiotic treatment successfully restricting growth and metastasis of mammary tumours in a mouse model, where the mice were inoculated with *F. nucleatum* (Ref. 64).

#### *Fusobacterium nucleatum* and chemotherapy resistance

As chemoresistance in BC is not yet fully understood, understanding mechanisms underlying drug resistance is vital to improve therapeutic approaches and clinical outcomes. Importantly, *F. nucleatum* has been reported to contribute to chemoresistance within CRC, ESCC and OSCC (Refs 122, 123, 124, 125).

In CRC cell lines, *F. nucleatum* was shown to promote chemoresistance to oxaliplatin and 5-fluorouracil (5-FU) by upregulating autophagy (Ref. 124) in a TLR4- and MYD88-dependent signalling pathway, and by preventing apoptosis via upregulation of ANO1 (Ref. 126) or BIRC3 (Ref. 125). Additionally, *F*. *nucleatum* promotes chemoresistance to 5-FU as well as cisplatin and docetaxel in ESCC (Refs 116, 122, 127) via upregulation of autophagy and preventing apoptosis. It is important to note that 5-FU is often used in BC treatment as a part of the FEC regime (5-FU, epirubicin and cyclophosphamide), in combination with docetaxel. Additionally, cisplatin is used in the neo-adjuvant setting for TNBC treatment (Ref. 128). Furthermore, *F. nucleatum* induced autophagy is linked to CRC metastasis (Ref. 70). These studies correlate with the observed poor patient response to neoadjuvant chemotherapy in ESCC tumours with high abundance of *F. nucleatum* (Refs 129, 130). Similarly, *F. nucleatum* was also shown to be enriched in OSCCs which were unresponsive to chemotherapy (Ref. 123).

#### *Fusobacterium nucleatum* and radiotherapy resistance

Serna *et al*. (Ref. 131) showed that chemotherapy and radiotherapy treatment was able to shift rectal adenocarcinoma tumours from *F. nucleatum*-positive to *F. nucleatum*-negative, which then showed improved relapse-free survival. However, any persistent *F. nucleatum* positivity correlated with a higher risk of relapse development.

Additionally, Dong *et al*. (Ref. 132) demonstrated that oral administration of *F. nucleatum* in CRC mice impaired the efficiency of radiotherapy, promoted colonic inflammation, increased the volume and number of tumours present and further increased metastases.

With radiotherapy being a major adjuvant therapy for eradication of BCs, *F. nucleatum* within the tumour tissue may be an important biomarker that predicts treatment response to radiotherapy.

#### *Fusobacterium nucleatum* and immunotherapy

Immune checkpoint therapy inhibits the interaction between a T-cell inhibitory receptor and its canonical ligand(s), allowing T lymphocytes to elicit antitumour responses (Ref. 133). For example, programmed cell death protein 1 (PD-1) when bound to its ligand PD-L1 inhibits T-cell activation (Ref. 134). While BC is considered to be less sensitive to immunotherapy than other cancers (Refs 135, 136, 137), PD-L1 is still expressed on a small subset of BC tumour cells (Refs 138, 139), and is associated with TNBC and HER2 overexpressing BCs (Refs 139, 140). Furthermore, treatment with ICIs such as atezolizumab has been approved for metastatic TNBC, and pembrolizumab improved clinical outcome for metastatic TNBC and high-risk early-stage TNBC (Refs 141, 142, 143, 144, 145). Recently, the FDA has granted accelerated approval to pembrolizumab in combination with chemotherapy for high-risk early-stage TNBC and for metastatic TNBC whose tumours express PD-L1. Therefore, the impact that *F. nucleatum* has on altering response to immunotherapy across BC subgroups should be further investigated, as well as its potential as a biomarker able to identify patients which will benefit from it.

In both patients and mice with CRC, Gao *et al*. found that *F. nucleatum* presence was correlated with improved response to PD-1/PD-L1 blockade treatment (Ref. 146). In the murine model of CRC, treatment with *F. nucleatum* enhanced anti-PD-L1 treatment response, and further improved survival (Ref. 146). Moreover, when *F. nucleatum* treatment was combined with anti-PD-L1 treatment, there was a significant increase in the amount of CD8+ T lymphocytes in the TME. Cancers with higher populations of CD8+ T lymphocytes are expected to have the greatest response to immunotherapy (Ref. 147). Therefore, it is possible to hypothesise that the alterations induced by *F. nucleatum* in CRC may result in a TME which responds more effectively to immunotherapy. However, a higher abundance of *F. nucleatum* in the patient's airways has been associated with a worse response of lung cancer to PD-1 blockade treatment (Ref. 148).

## Conclusions and future directions

*F. nucleatum* has been identified as a bacterial species which colonises the breast and recent findings indicate that it may contribute to BC progression and metastatic development (Ref. 64). However, the underlying pathogenic mechanisms are poorly understood, with few studies investigating the potential role of *F. nucleatum* in BC patient cohorts. Typically, *F. nucleatum* has been identified in approximately 20–30% of BC tumours (Refs 10, 29, 64), but correlation with clinical characteristics such as tumour stage or BC subgroup requires further investigation.

The literature from research into other cancer types, including CRC, indicates that *F. nucleatum* is able to modulate the local TME, promoting an inflammatory state and further interacting with and influencing infiltrating immune cells. The question of whether the presence of *F. nucleatum* in the TME of breast carcinomas will show the same trends in inflammation and immunomodulation requires further investigation. In particular, advanced *in vitro* models such as organoids could be beneficial to recapitulate how the hypoxic environment of the tumour influences the survival and growth of the anaerobic *F. nucleatum.* Additionally, *in vivo* models should be considered for further investigating the relationship between *F. nucleatum* in breast tumours with the tumour immune microenvironment (Ref. 64).

Multiple protocols have been suggested in order to quantify the presence of *F. nucleatum* in cancer patients, for example, a faecal *F. nucleatum*-based assay for CRC (Ref. 149), and qPCR of *F. nucleatum* DNA in tumour tissue (Refs 50, 150, 151, 152, 153). However, current literature highlights the difficulties in detecting microbial DNA from human host tissues, which is exacerbated in low microbial biomass tumour tissues such as is seen in the breast (Refs 35, 154, 155, 156). Before *F. nucleatum* can be used as a biomarker for any cancer type, a sensitive, yet cost-effective assay must be developed to detect and quantify *F. nucleatum* in patients. Salivary *F. nucleatum* DNA has been identified as a non-invasive biomarker for CRC and gastric cancer diagnosis (Refs 53, 157). Further research is required to determine if these findings could also apply to other *F. nucleatum*-linked cancers, including breast.

Targeting *F. nucleatum* in the tumour could potentially introduce an exciting novel treatment option. Parhi *et al*. (Ref. 64) showed that antibiotic treatment of a BC mouse model inoculated with *F. nucleatum* eliminated *F. nucleatum* from the tumour and further suppressed *F. nucleatum*-induced tumour growth. It is therefore tempting to consider antibiotics adjunct to current BC treatments to target tumour-promoting bacteria. However, given the role of the patient's microbiome in influencing drug efficacy (Refs 12, 35, 37, 38, 158, 159, 160), broad microbe-targeting treatments may not be beneficial. Interestingly, a *F. nucleatum*-specific bacteriophage, FNU1, has been recently suggested as a means to eradicate the oncobacterium from the tumour (Ref. 161). Strong evidence supports the influence of the gut microbiome in response to cancer therapy, most notably ICIs (Ref. 162). Given the increasing use of ICIs in BC, especially for TNBC (Refs 141, 142, 143, 163), the potential interaction between *F. nucleatum* within the breast and ICI therapy (Ref. 146) is an especially interesting area of future research.

In conclusion, by better understanding the consequences of the presence of this bacterium, it will provide valuable insights into the role of the microbiota in BC progression and how it influences treatment efficacy in patients.
